# Pulmonary Vascular Disease and Cardiopulmonary Exercise Testing

**DOI:** 10.3389/fphys.2020.00964

**Published:** 2020-08-05

**Authors:** Pierantonio Laveneziana, Jason Weatherald

**Affiliations:** ^1^Sorbonne Université, INSERM, UMR S1158 Neurophysiologie Respiratoire Expérimentale et Clinique, Paris, France; ^2^AP-HP, Groupe Hospitalier Universitaire APHP-Sorbonne Université, Sites Pitié-Salpêtrière, Saint-Antoine et Tenon, Service des Explorations Fonctionnelles de la Respiration, de l’Exercice et de la Dyspnée (Département R3S), Paris, France; ^3^Division of Respirology, Department of Medicine, University of Calgary, Calgary, AB, Canada; ^4^Libin Cardiovascular Institute of Alberta, University of Calgary, Calgary, AB, Canada

**Keywords:** cardiopulmonary exercise testing, dyspnea, prognosis, pulmonary hypertension, dynamic hyperinflation, ventilatory inefficiency

## Abstract

Cardiopulmonary exercise testing (CPET) is of great interest and utility for clinicians dealing Pulmonary Hypertension (PH) in several ways, including: helping with differential diagnosis, evaluating exercise intolerance and its underpinning mechanisms, accurately assessing exertional dyspnea and unmasking its underlying often non-straightforward mechanisms, generating prognostic indicators. Pathophysiologic anomalies in PH can range from reduced cardiac output and aerobic capacity, to inefficient ventilation, dyspnea, dynamic hyperinflation, and locomotor muscle dysfunction. CPET can magnify the PH-related pathophysiologic anomalies and has a major role in the management of PH patients.

## Introduction

Pulmonary arterial hypertension (PAH) is characterized by anomalies in pulmonary arteries (abnormal proliferation of smooth muscle and endothelial cells) which results in cardiovascular anomalies such as increase in pulmonary vascular resistance (PVR) and finally right ventricular failure (Galie et al., 2015; Humbert et al., 2019; Simonneau et al., 2019). PAH may present with non-specific symptoms and signs such as generalized fatigue, limitation of daily-activities and dyspnea, and this may prevent clinicians from diagnosing it early in the course of PAH and thus most of the time the diagnosis is made at the time of advanced right heart failure. Right-heart catheterization (RHC) is fundamental to confirm the diagnosis of PAH (Simonneau et al., 2019) and recently a new hemodynamic definition of PAH has been proposed (a mean pulmonary artery pressure >20 mmHg instead of previous one ≥25 mmHg) based on the analysis of large databases (Kovacs et al., 2017) and a meta-analysis of normal hemodynamics (Kovacs et al., 2009) in order to identify patients with early pulmonary vascular disease (Simonneau et al., 2019).

Cardiopulmonary exercise testing (CPET) is of great interest and utility for clinicians dealing PH in evaluating exercise intolerance and its underpinning mechanisms, accurately assessing exertional dyspnea and unmasking its underlying mechanisms, which are often not straightforward. Previous studies have shown that PAH management at an early stage of the disease translates into better outcomes (Galie et al., 2008; Humbert et al., 2011; Lau et al., 2015). Therefore, it appears crucial to establish early diagnosis and CPET can help clinicians in the differential diagnosis and evaluating prognosis in such an especially fragile population.

## Pathophysiologic Response-Profile to Exercise in Pulmonary Hypertension

**FIGURE 1:**
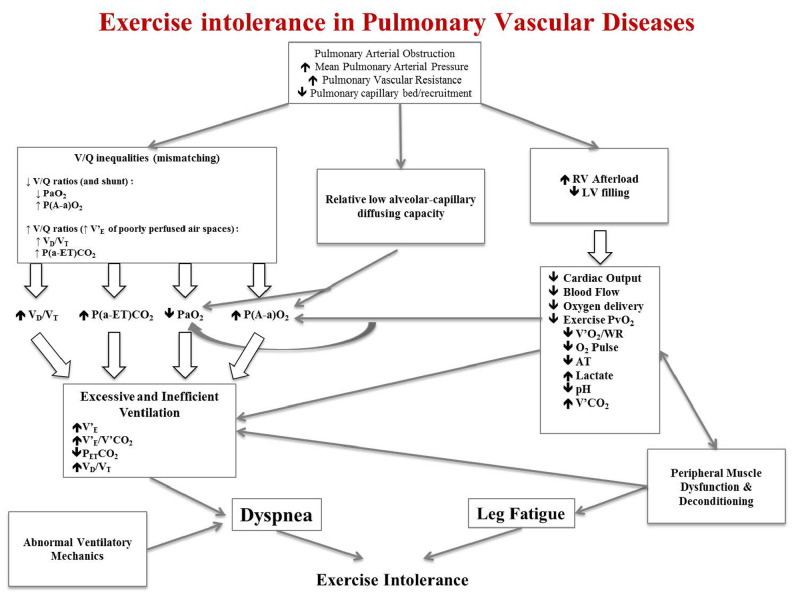
Schematic pathophysiologic pathway leading to exercise intolerance and exertional dyspnea in pulmonary hypertension. RV, right ventricle; LV, left ventricle; V’_E_, minute ventilation; V’_E_/V’CO_2_, ratio of minute ventilation to carbon dioxide production; P_ET_CO_2_, end-tidal pressure of carbon dioxide; V_D_/V_T_, dead space to tidal volume fraction; V’O_2_, oxygen consumption; WR, work rate; O_2_ pulse, V’O_2_ to heart rate (HR) ratio; V’CO_2_, carbon dioxide production; PvO_2_, venous pressure of oxygen; PaO_2_, arterial partial pressure of oxygen; PaCO_2_, arterial partial pressure of carbon dioxide. This is an original figure, no permission is required.

CPET can magnify the PH-related pathophysiologic anomalies and has a major role in the management of PH patients. Pathophysiologic anomalies in PH can widely range from reduced cardiac output and aerobic capacity, to pulmonary gas exchange and ventilatory efficiency anomalies, dyspnea, dynamic hyperinflation and locomotor muscle dysfunction (Figure 1).

Pulmonary vascular obstruction along with concurrent increased mean PAP and PVR and reduced pulmonary capillary bed and recruitment give rise to three different pathophysiologic anomalies: (1) ventilation/perfusion (V/Q) inequalities; (2) pulmonary gas exchange anomalies; (3) increased right ventricle (RV) afterload and concomitant reduced left ventricle (LV) filling. These three major pathophysiologic derangements are responsible of characteristic anomalies observed during CPET that can ultimately explain exertional dyspnea and exercise intolerance (Figure 1).

V/Q inequalities can manifest with either low V/Q ratios and shunt (right to left shunt through a patent foramen ovale, for example) or high V/Q ratios caused by increased minute ventilation (V’_E_) of poorly perfused air spaces (Oudiz et al., 2010) V/Q mismatching can result in hypoxemia (reduced arterial partial pressure of oxygen, PaO_2_), high dead space to tidal volume fraction (V_D_/V_T_) and widening of the alveolar-arterial pressure difference of oxygen [P(A-a)O_2_] and of the arterial-end-tidal pressure difference of carbon dioxide [P(a-ET)CO_2_]. These anomalies can stimulate an excessive V’_E_ response to exercise along with altered chemosensitivity and inefficient ventilation mirrored by the increased steepness with which V’_E_ rises with respect to CO_2_ production (V’CO_2_) (i.e., increased V’_E_/V’CO_2_ slope) (D’Alonzo et al., 1987; Riley et al., 2000; American Thoracic Society, 2003; Velez-Roa et al., 2004; Naeije and van de Borne, 2009; Wensel et al., 2009; Laveneziana et al., 2013b; Farina et al., 2018; Weatherald et al., 2020). Inefficient ventilation and altered chemosensitivity translate into increase ventilatory demand, V’_E_/V’CO_2_ and V_D_/V_T_, decrease end-tidal pressure of carbon dioxide (P_ET_CO_2_) and hypocapnia (reduced arterial partial pressure of carbon dioxide, PaCO_2_) (Riley et al., 2000; Yasunobu et al., 2005; Zhai et al., 2011; Scheidl et al., 2012; Godinas et al., 2017; Weatherald et al., 2020).

The reduced pulmonary capillary bed and recruitment at rest can be amplified during CPET and translated in pulmonary gas exchange anomalies such as a relative low alveolar-capillary diffusing capacity; this can be mirrored by a reduced diffusing capacity or transfer factor of the lung for carbon monoxide (DLCO or TLCO) at rest and a reduced PaO_2_ with enlargement of P(A-a)O_2_ during CPET.

Impaired cardiac function (due to increased RV afterload and concomitant reduced LV filling) along with peripheral muscle dysfunction and deconditioning (Bauer et al., 2007; Tolle et al., 2008; Mainguy et al., 2010; Dimopoulos et al., 2013) result in reduced cardiac output and blood flow to the periphery. This translates to reduced oxygen delivery to working locomotor muscles and reduced venous pressure of oxygen (PvO_2_), which results in reduced aerobic capacity with attendant reduced anaerobic threshold (AT) and oxygen consumption (V’O_2_). Reduced oxygen delivery also causes early onset of lactic acidosis and increased V’CO_2_, which further contributes to the excessive V’_E_ response to CPET (Nootens et al., 1995; Sun et al., 2001; Deboeck et al., 2004; Hasler et al., 2016; Naeije and Badagliacca, 2017; Weatherald et al., 2018a). The reduced mixed venous O_2_ content from altered cardiac output can also contribute and amplify exertional hypoxemia.

Mechanical anomalies on tidal volume (V_T_) expansion and dynamic lung hyperinflation can also play a crucial role into the genesis of exertional dyspnea and therefore exercise intolerance (Richter et al., 2012; Laveneziana et al., 2013b, 2015; Manders et al., 2016; Boucly et al., 2020), and can be easily detected during CPET (Laveneziana et al., 2013b, 2015; Boucly et al., 2020).

## Peripheral Muscle Dysfunction

Deconditioning and peripheral muscle abnormalities are important contributors to exercise intolerance. In chronic heart failure, which shares similar limitations in cardiac output reserve as PAH and CTEPH, oxygen transport and diffusion at the level of skeletal muscle are abnormal (Esposito et al., 2010). Tissue oxygen saturation, oxygen extraction and muscle microcirculatory function may be impaired to an even greater degree in PAH compared with left heart failure (Tolle et al., 2008; Dimopoulos et al., 2013). Peripheral muscle in PAH patients is structurally and functionally abnormal, with a lower relative proportion of type I fibers and reduced quadriceps, forearm, and respiratory muscle strength compared to controls, which may be an important determinant of low peak V’O_2_ (Bauer et al., 2007; Mainguy et al., 2010).

Respiratory muscle strength has also been shown to be about 40% lower in CTEPH patients (Manders et al., 2016). The mechanism of generalized skeletal muscle dysfunction in PAH may be a result of microcirculation rarefaction and an imbalance in angiogenic factors (Potus et al., 2014). Improvements in exercise capacity with exercise training in individuals with heart failure or peripheral vascular disease (Duscha et al., 2011) have been linked to improvements in skeletal muscle microcirculatory density, capillary-to-fiber ratio and mitochondrial volume (Esposito et al., 2011), which may be mechanisms by which training can improve exercise capacity in stable patients with PAH (Mereles et al., 2006; Ehlken et al., 2016). Peripheral muscle dysfunction is a potential relevant e hidden factor that can worsen the prognosis of PAH patients. Recently, Valli et al. have pointed out that patients with PAH may present with less efficient muscular oxygen utilization than healthy controls. Notably high energy expenditure had a relevant independent prognostic impact (Valli et al., 2019).

## Translating PH-Related Pathophysiologic Anomalies Into CPET Variables: Exploring Factors Explaining Exercise Limitation in PH

One of the indications of CPET is to explore the underlying mechanisms of exertional dyspnea and to detect mechanisms of exercise intolerance. Table 1 summarizes the main CPET-derived variables defining ventilatory, respiratory mechanical, cardiovascular and pulmonary vascular limitation accompanied or not by gas exchange anomalies to exercise.

**TABLE 1:** Variables defining ventilatory and respiratory mechanical limitation (left panel) accompanied or not by gas exchange anomalies to exercise, and variables defining cardiovascular and pulmonary vascular limitation (right panel) accompanied or not by gas exchange anomalies to exercise.

**Ventilatory and respiratory mechanical limitation to exercise**	**Cardiovascular and pulmonary vascular limitation to exercise**
BR < 15–20%	BR > 15–20%
Dynamic Hyperinflation (decrease in IC > 140 mL)	V’O_2_/HR < 70%
V_T_ plateau	V’O_2_/Work Rate↓
RR > 50–55 breaths/min (if restrictive pattern)	Flat (and declining) V’O2/HR trajectory
V_T_ = IC or > 60% VC (if restrictive pattern)	Abnormal HR/V’O_2_ slope (>50)
HR peak < HR predicted	Chronotropic incompetence
EILV >90% TLC at peak exercise	Abnormal blood pressure response to exercise
V_T_/IC >70% at peak exercise	ECG abnormalities during exercise
The tidal inspiratory flow >50 to 70% maximal inspiratory flow (in health <50–70%)	
**With or without**	**With or without**
Gas exchange anomalies:	Gas exchange anomalies:
V_D_/V_T_ ↑	V_D_/V_T_ ↑
P(A-a)O_2_ ↑	P(A-a)O_2_ ↑
Decrease of PaO_2_ ≥ 10 mmHg	Decrease of PaO_2_ ≥ 10 mmHg
Decrease of SpO_2_ ≥ 5% and/or SpO_2_ peak ≤ 88%	Decrease of SpO_2_ ≥ 5% and/or SpO_2_ peak ≤ 88%
PaCO_2_ peak > 45–50 mmHg	

*BR, breathing reserve; IC, Inspiratory Capacity; VT, tidal volume; RR, Respiratory Rate; VC, Vital Capacity; HR, Heart Rate; EILV, End-Inspiratory Lung Volume; VD/VT, dead space to tidal volume fraction; P(A-a)O_2_, alveolar-arterial pressure difference of oxygen;, PaO_2_, partial pressure of oxygen; SpO_2_, pulse oximetry saturation; PaCO_2_, arterial partial pressure of carbon dioxide; V’O_2_, oxygen consumption; P(a-ET)CO_2_, arterial-end-tidal pressure difference of carbon dioxide; ↓, reduced; ↑, increased.*

Two variables are used to detect exercise intolerance (Radtke et al., 2019) peak V’O_2_ during an incremental CPET has well defined normal values (Puente-Maestu et al., 2018) and V’O_2_ at the anaerobic/ventilatory threshold (AT) has the advantage of being an effort independent measure of exercise tolerance (Agusti et al., 1997; ATS/ACCP, 2003; Palange et al., 2007; Puente-Maestu et al., 2016; Huckstepp et al., 2018). The disadvantage is that it relies on pattern recognition for accurate detection (which may differ from operator to operator due to lack of experience or inaccurate detection related to software - never trust software for detecting AT! - or wrong pattern recognition) and some severely impaired PH patients may not be able to attain the AT despite a good effort.

Cardiovascular limitation to exercise is not straightforward and may be defined by certain interrelated variables (Table 1). A reduced slope or late plateau of the V’O_2_ trajectory (i.e., a reduced V’O_2_/work rate relationship ≤8), or plateau (early or late during exercise) of the oxygen pulse (V’O_2_ to heart rate ratio, i.e., V’O_2_/HR), or an abnormal HR/V’O_2_ slope (>50) may be typical (Palange et al., 2018).

Pulmonary vascular limitation to exercise is not straightforward as well and may rely on evidence of increased V’_E_/V’CO_2_ slope and ratio at AT in addition to the above-mentioned anomalies (Weatherald et al., 2018b). Other typical features of pulmonary vascular disease are low levels of P_ET_CO_2_ at AT, a V_D_/V_T_ which remains stable or increases or fails to decrease from baseline, a P(a-ET)CO_2_ which fails to became negative during exercise and, sometimes, a P(A-a)O_2_ which widens on exertion (Weatherald et al., 2020; Table 1). Of note, it should be pointed out that the finding of high V’_E_/V’CO_2_ at AT (≥34–35) and low P_ET_CO_2_ at AT (≤30 mmHg) without an alternative explanation in patients presenting with unexplained dyspnea and exercise limitation should prompt further diagnostic testing to exclude PAH or CTEPH (Weatherald et al., 2018b) particularly in those patients with risk factors, such as prior venous thromboembolism, systemic sclerosis or a family history of PAH. These gas exchange anomalies are usually not found in patients with pulmonary venous hypertension secondary to cardiac diseases (Weatherald et al., 2018b). Associated low level of hemoglobin will enhance oxygen flow deficiency. Ischemic heart disease or cardiomyopathy may present with electrocardiographic or blood pressure anomalies during CPET (Palange et al., 2018).

**FIGURE 2:**
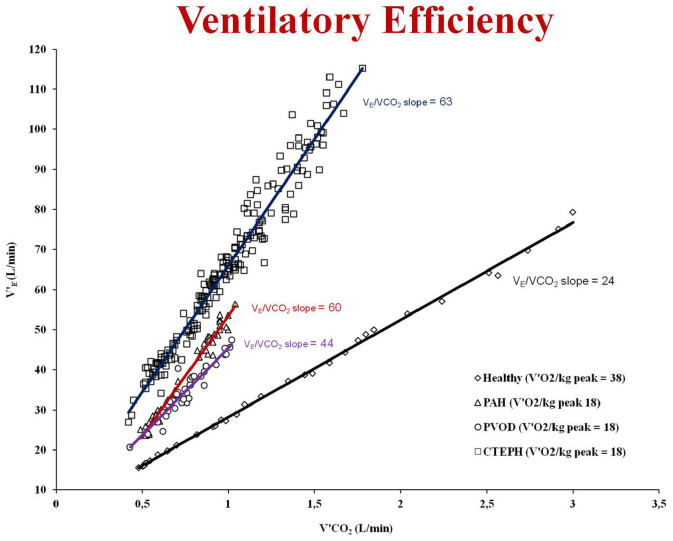
Examples of ventilatory efficiency (V’_E_/V’CO_2_ slope) in a healthy subject with a peak oxygen uptake (V’O_2_) of 38 mL/Kg/min (black line and rhomboid), in a patient with pulmonary veno-occlusive disease (PVOD) with a peak V’O_2_ of 18 mL/Kg/min (violet line and circles), in a patient with pulmonary arterial hypertension (PAH) with a peak V’O_2_ of 18 mL/Kg/min (red line and triangles) and in a patient with chronic thromboembolic pulmonary hypertension (CTEPH) with a peak V’O_2_ of 18 mL/Kg/min (blue line and squares). This is an original figure, no permission is required.

Pulmonary gas exchange limitation to exercise is not straightforward as well and may rely on evidence of inefficient carbon dioxide exchange which can be signaled by high V_D_/V_T_ and often by high exercise V’_E_/V’CO_2_ (Figure 2) or (alone or in combination with) inadequate oxygen exchange signaled by low PaO_2_ or, less directly, by desaturation at pulse oximetry.

Elevated V’_E_/V’CO_2_ (ventilatory inefficiency) and reduced resting PaCO_2_ (hypocapnia) are frequent in pulmonary vascular disease such as PAH, chronic thromboembolic pulmonary hypertension (CTEPH) and pulmonary veno-occlusive disease (PVOD) (Weatherald et al., 2018b) and correlate with negative prognosis (Hoeper et al., 2007; Deboeck et al., 2012; Schwaiblmair et al., 2012; Groepenhoff et al., 2013). Abnormally reduced resting PaCO_2_ signals augmented chemosensitivity or an abnormal PaCO_2_ set-point. A low resting PaCO_2_ predicts a worse prognosis in PAH (Hoeper et al., 2007). However, high V_D_/V_T_ does not cause low resting PaCO_2_, therefore an altered PaCO_2_ set-point, increased neural respiratory drive, and/or increased chemosensitivity must explain hypocapnia and consequently, the high V’_E_/V’CO_2_ slope. Several factors such as metabolic acidosis, hypoxemia, baroreceptors in the pulmonary vessels and abnormal activation of the sympathetic nervous system have an influence on the PaCO_2_ set-point (Wasserman et al., 1975; Whipp and Ward, 1998; Sun et al., 2002; Velez-Roa et al., 2004; Wensel et al., 2004; Laveneziana et al., 2013b; Weatherald et al., 2018c). The evaluation of chemosensitivity and/or the PaCO_2_ set-point during exercise is difficult and can be problematic. Autonomic dysfunction, increased sympathetic nervous system activity, and an altered CO_2_ set-point relate to chemoreflex sensitivity. Recently Farina et al. (2018) performed minute-to-minute blood gas analysis during exercise in 18 patients with pulmonary vascular disease. They run hypoxic and hypercapnic challenge tests to assess peripheral and central chemosensitivity and found an increase in chemoreceptor sensitivity in both PAH and CTEPH that did not correlate (the peripheral chemoreceptor responses to hypoxia and hypercapnia) with any exercise variables. The “non-invasive” evaluation of the PaCO_2_ set-point during exercise is extremely difficult; one method is to assess the maximal end-tidal CO_2_ pressure (maximal P_ET_CO_2_) value between the AT and respiratory compensation point where P_ET_CO_2_ is constant and, therefore, is supposed to truly reflect the real PaCO_2_ set-point (Agostoni et al., 2002; Agostoni et al., 2010; Laveneziana et al., 2010). Recently, Weatherald et al. have pointed out that patients with resting hypocapnia (PAH, *n* = 34; CTEPH, *n* = 19; PVOD, *n* = 6) had worse cardiac function and more severe gas exchange anomalies during CPET (Weatherald et al., 2020). High chemosensitivity and an altered PaCO_2_ set-point were likely explanations for resting hypocapnia and high V’_E_/V’CO_2_ on exertion. The PaCO_2_ set-point, estimated by the maximal P_ET_CO_2_ was the strongest correlate of peak exercise capacity and V’_E_/V’CO_2_, suggesting that this variable could be used as a non-invasive measure of disease severity even during submaximal exercise (Weatherald et al., 2020). Taken together, the results of the two studies from Weatherald et al. (2020) and Farina et al. (2018) imply that hypocapnic patients and/or those with low maximal P_ET_CO_2_ during exercise have autonomic dysfunction and a lower CO_2_ set-point. Thus, resting PaCO_2_ or maximal P_ET_CO_2_ on exertion could be used to identify patients with probably autonomic dysfunction or to help develop future studies that target the sympathetic nervous system in pulmonary vascular disease.

**FIGURE 3:**
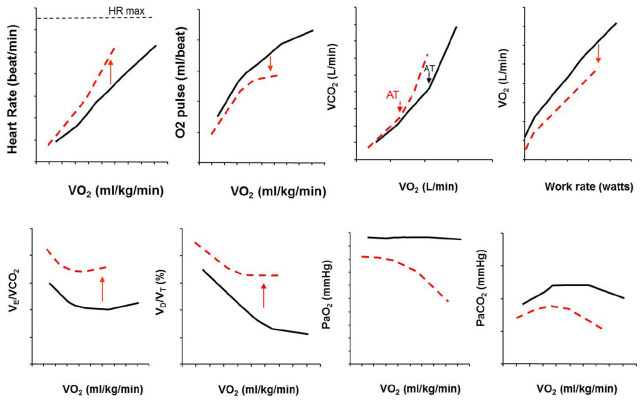
Typical exercise response profile of a PAH patient (red dotted line) compared with healthy control (black line) undergoing maximal incremental symptom-limited CPET. Abbreviations in the text. This is an original figure, no permission is required.

Ventilatory limitation to exercise can also be detected in some PH patients during CPET (Table 1). Beside the well-known breathing reserve, i.e., the comparison of peak V’_E_ to MVV (maximal voluntary ventilation), other indicators of ventilatory limitation to exercise can be appreciated: constraints on dynamic V_T_ expansion relative to resting or dynamic decrease in inspiratory capacity (IC) used also to appreciate a critical reduction in inspiratory reserve volume (IRV) (Table 1; Laveneziana et al., 2013b, 2015; Boucly et al., 2020). More recently, evidence of ventilatory limitation has been suggested by the occurrence of important expiratory flow limitation (EFL) >25% at peak exercise (Johnson et al., 1999; Palange et al., 2007; Puente-Maestu et al., 2016; Huckstepp et al., 2018) and some other indicators of mechanical ventilatory limitations to exercise such as end-inspiratory lung volume (EILV) >90% TLC alone or in combination with V_T_/IC >70% at peak exercise have recently been observed in some patients with pulmonary vascular disease (Table 1). Figure 3 represents the typical exercise response profile of a PAH patient undergoing maximal incremental symptom-limited CPET. Table 2 summarizes the main alterations that can be observed in patients with pulmonary vascular disease during CPET.

**TABLE 2:** Typical CPET anomalies in patients with pulmonary vascular diseases.

		**PAH**	**CTEPH**	**PVOD**
Metabolic and cardiovascular	Peak V’O_2_	↓	↓	↓
	V’O_2_ at AT	↓	↓	↓↓
	V’O_2_/WR	↓	↓	↓
	O_2_ pulse	↓	↓	↓
Ventilation and mechanics	Peak V’_E_	↓	↓	↓
	Breathing Reserve	Normal	Normal	Normal
	Dynamic hyperinflation	Possible	Possible	?
Gas exchange	V’_E_/V’CO_2_ slope	↑	↑↑	↑↑
	V’_E_/V’CO_2_ at AT	↑	↑↑	↑↑
	P_ET_CO_2_	↓	↓↓	↓↓
	SaO_2_	↓	↓↓	↓↓
	P_a–ET_CO_2_	↑	↑↑	↑↑
	P_A–a_O_2_	↑	↑↑	↑↑
	V_D_/V_T_	↑	↑↑	↑↑

*CPET, cardiopulmonary exercise testing; PAH, pulmonary arterial hypertension; CTEPH, chronic thromboembolic pulmonary hypertension; PVOD; pulmonary veno-occlusive disease; V’O_2_, oxygen consumption; AT, ventilator/anaerobic threshold; WR, work rate; O_2_ pulse, peak V’O_2_ to heart rate ratio at peak exercise; V’E, minute ventilation; V’E/V’CO_2_, ratio of minute ventilation to carbon dioxide production (V’CO_2_); PETCO_2_, end-tidal pressure of carbon dioxide; SaO_2_, arterial oxygen saturation; PA-aO_2_, alveolar-arterial oxygen pressure gradient at peak exercise; Pa-ETCO_2_, arterial to end-tidal carbon dioxide pressure gradient at peak exercise; VD/VT, physiologic dead space fraction as ratio of dead space (VD) to tidal volume (VT) at peak exercise.*

## Mechanisms Explaining Exertional Dyspnea in PH

**FIGURE 4:**
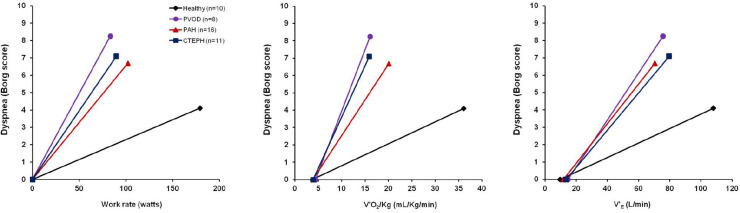
Exertional dyspnea intensity as measured by Borg score is displayed in response to increasing work rate **(left panel)**, increasing oxygen consumption (V′_2_/Kg, **mid panel**) and increasing minute ventilation (V’_E_, **right panel**) during symptom limited cardiopulmonary exercise testing in 10 healthy subjects (black line and rhomboid), 8 patients with pulmonary veno-occlusive disease (PVOD) (violet line and circles), 16 patients with pulmonary arterial hypertension (PAH) (red line and triangles), 11 patients with chronic thromboembolic pulmonary hypertension (CTEPH) (blue line and squares). The origin of the data provided in Figure 4 is from Laveneziana et al. (2013b, 2014a, 2015), Boucly et al. (2020) and Weatherald et al. (2020).

Exertional dyspnea is most frequent and cumbersome symptom in patients with idiopathic PAH, CTEPH, and PVOD (Laveneziana et al., 2013b, 2014a, 2015; Laviolette and Laveneziana, 2014; Boucly et al., 2020; Figure 4).

**FIGURE 5:**
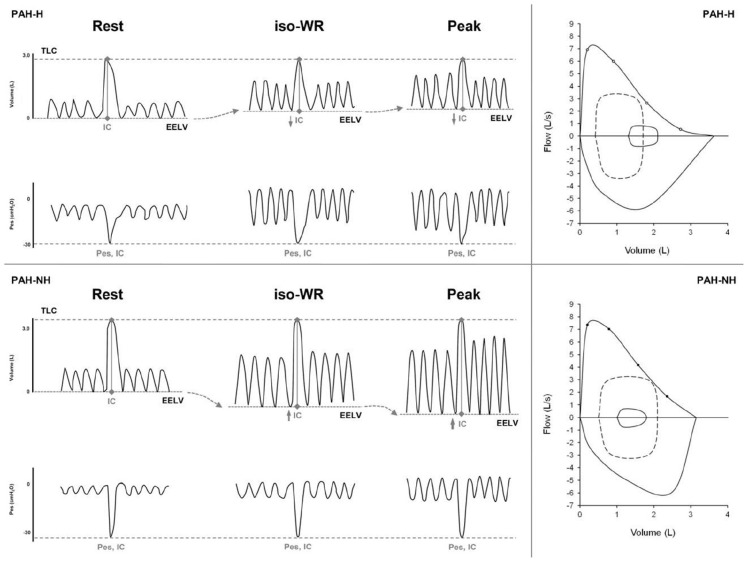
Tracings of lung volume (Volume) and esophageal pressure (Poes) from inspiratory capacity (IC) maneuvers taken during resting breathing, at 60 watts (iso-WR) and peak-exercise from one representative PAH patient who reduced IC (or increased end- expiratory lung volume, i.e., EELV) during exercise [PAH hyperinflator (PAH-H), upper left panel] and one who increased IC (or reduced EELV) [PAH non-hyperinflator (PAH-NH), lower left panel]. Please note that, regardless of changes in IC during exercise, dynamic peak inspiratory Poes recorded during IC maneuvers (Poes, IC) is remarkably preserved in both PAH-H (upper left panel) and PAH-NH (lower left panel). Maximal and tidal flow-volume loops (average data) are shown at rest and at peak- exercise in PAH-H (upper right panel) and PAH-NH (lower right panel). Tidal flow-volume loops are provided at rest (solid line) and at peak-exercise (dashed line). Note a significant decrease in dynamic IC during exercise in PAH-H compared with PAH-NH. TLC, total lung capacity. Reproduced with permission of Laveneziana et al. (2015).

Although researchers have worked hard to try to explain this symptom, its underpinning mechanisms remain at present not completely understood (Laveneziana et al., 2013b, 2014a, 2015; Laviolette and Laveneziana, 2014; Boucly et al., 2020). Previous research has particularly emphasized the cardio-and-pulmonary-vascular factors contributing to exertional dyspnea (Sun et al., 2001) by pointing out the effects of combined impaired cardiac function and abnormal pulmonary gas exchange on exertion as the result of primary anomalies of pulmonary vessels on the increased ventilatory drive and therefore on the resultant exertional dyspnea (Sun et al., 2001). Nonetheless, augmented ventilatory drive cannot alone explain the origin of the multifaceted symptom of dyspnea, and other contributions stemming from respiratory and skeletal muscle (dys)function, as well as psychological and emotional status may come into play. Recently, abnormalities of breathing mechanics have been pointed out in some PAH and CTEPH patients during exercise (Richter et al., 2012; Laveneziana et al., 2013b, 2015; Dorneles et al., 2019; Boucly et al., 2020; Figure 5) and are likely to precipitate exertional dyspnea in these two populations (Laveneziana et al., 2013b, 2015; Dorneles et al., 2019; Boucly et al., 2020; Figure 4).

**FIGURE 6:**
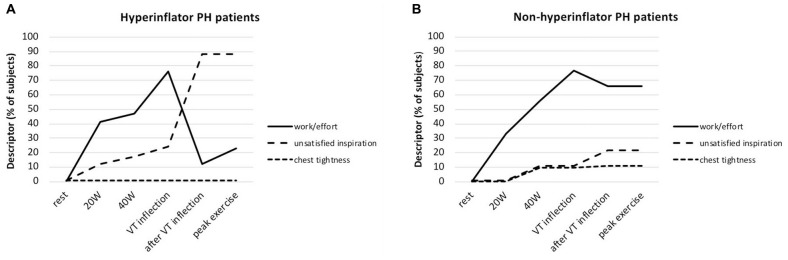
**(A)** Selection frequency of the three descriptor phrases evaluated during symptom-limited incremental cycle exercise in patients with pulmonary hypertension (PH) who hyperinflate (Hyperinflators) during exercise: increased work/effort, unsatisfied inspiration, and chest tightness. Data are presented as mean at rest, at 20W (iso-WR 1), at 40 W (iso-WR 2), at the tidal volume (VT) inflection point, after the VT inflection point and at peak exercise. **(B)** Selection frequency of the three descriptor phrases evaluated during symptom-limited incremental cycle exercise in patients with pulmonary hypertension (PH) who deflate (Non-hyperinflators) during exercise: increased work/effort, unsatisfied inspiration, and chest tightness. Data are presented as mean at rest, at 20W (iso-WR 1), at 40 W (iso-WR 2), at the tidal volume (VT) inflection point, after the VT inflection point and at peak exercise. Reproduced with permission of Boucly et al. (2020).

Now, what kind of abnormalities of breathing mechanics have been observed in PAH and CTEPH patients that can explain, at least in part, dyspnea generated during exertion and during laboratory-based CPET? Without giving to much of details on the underlying mechanisms of the anomalies of breathing mechanics encountered during CPET in PAH and CTEPH patients (which goes outside the scope of this review), we can say that some features are the development of EFL and dynamic lung hyperinflation (indicated by an increased end- expiratory lung volume, i.e., EELV that is mirrored by a decrease of the same amount/proportion in IC on exertion) with concurrent limitation of V_T_ expansion and attainment of a critical IRV in at least 60% of these patients (Richter et al., 2012; Laveneziana et al., 2013b, 2015; Dorneles et al., 2019; Boucly et al., 2020; Figure 5). Of course some considerations must be made here: EFL is most of the time not present at rest and resting IC is preserved (Laveneziana et al., 2013b, 2015; Boucly et al., 2020), even in CTEPH pre and post-pulmonary endarterectomy (Richter et al., 2017); what is evident in 60% of these patients (PAH and CTEPH) is a reduction of the forced expiratory flow at low lung volumes (FEF_75%_) where V_T_ occurs; this predisposes to dynamic decrease in IC and limitation of V_T_ expansion with concomitant attainment of a critical IRV in some of these PAH and CTEPH patients, as it may occur in some patients with asthma (Laveneziana et al., 2006, 2013a), chronic obstructive pulmonary disease (COPD) (Laveneziana et al., 2011, 2014b; Guenette et al., 2012; Soumagne et al., 2016) and chronic heart failure (CHF) (Laveneziana et al., 2009; Laveneziana and Di Paolo, 2019; Smith et al., 2019). The sensory consequence of this is the escalation of dyspnea intensity and the transition in its qualitative description from “work/effort” to “unsatisfied inspiration” (Figure 6), as is the case in some asthmatics (Laveneziana et al., 2006, 2013a), and COPD patients (Laveneziana et al., 2011, 2014b; Guenette et al., 2012; Soumagne et al., 2016).

Of note, these particular PAH and CTEPH patients present also with a high level of anxiety which is frequently associated with dyspnea on exertion (Boucly et al., 2020). It should be noted here that 40% of these PAH and CTEPH patients do not manifest decrease in IC (meaning that they deflate normally during exercise), nor limitation of V_T_ expansion nor attainment of a critical IRV during CPET (Laveneziana et al., 2013b, 2015; Boucly et al., 2020). Dyspnea intensity in this group of PAH and CTEPH is less important than in the other group previously described (Laveneziana et al., 2013b, 2015; Boucly et al., 2020) and its qualitative description remains predominantly the sense of breathing “work/effort” (Laveneziana et al., 2013b, 2015; Boucly et al., 2020; Figure 6), as it occurs in healthy subjects on exertion (Laveneziana et al., 2013b, 2014b).

Another important point to bring to reader attention here is whether the dynamic decrease in IC observed during CPET in some PAH and CTEPH patients is truly reflective of dynamic lung hyperinflation or could be related to a dysfunction of inspiratory muscle (weakness or fatigue). The occurrence of fatigue or the overt presence of weakness of inspiratory muscle in PAH patients have been questioned by two studies from Laveneziana et al. (2013b, 2015; Figure 5) that have assessed the reliability of IC maneuvers in PH patients by (1) comparing inspiratory esophageal pressure (Poes) values during IC manoeuvers, (2) comparing sniff-Poes values pre- vs. post-exercise in PH patients and (3) comparing TLC pre- vs post-maximal CPET. These studies clearly pointed out that (1) Poes values measured during IC manoeuvers were remarkably preserved during exercise and were independent of exercise intensity and V’_E_ in PAH (Laveneziana et al., 2015), (2) sniff-Poes values were identical pre- vs. post-exercise in PH patients (Laveneziana et al., 2015) and (3) TLC pre CPET was superimposed to TLC immediately post-exercise in PH patients (Laveneziana et al., 2013b, 2015). Taken together these findings prove that IC maneuvers are reliable (Laveneziana et al., 2013b, 2015) and that inspiratory muscle dysfunction is unlikely to manifest, at least in these stable PAH patients (Laveneziana et al., 2013b, 2015; Figure 5).

## Prognostic Utility of CPET

There is good evidence that CPET variables can be used to measure disease severity and are predictive of survival and time to clinical worsening in PAH and CTEPH patients as well as potential treatment targets for PAH patients, with objectives of obtaining peak V’O_2_ >15 mL.min^–1^.kg^–1^ or > 65% predicted and a V’_E_/V’CO_2_ slope of <36 (Galie et al., 2015; Puente-Maestu et al., 2016). PAH patients with a peak V’O_2_ less than 11 mL.min^–1^.kg^–1^ or a V’_E_/V’CO_2_ slope ≥45 are considered high risk with an estimated 1-year mortality of >10% according to European Society of Cardiology/European Respiratory Society guidelines (Galie et al., 2015). Peak V’O_2_ and V’_E_/V’CO_2_ have been associated with survival in several studies including PAH and CTEPH patients (Wensel et al., 2002; Deboeck et al., 2012; Schwaiblmair et al., 2012; Groepenhoff et al., 2013). Wensel et al. (2013) demonstrated that peak V’O_2_ provides additional prognostic value to resting haemodynamics in patients with PAH. Those with a low V’O_2_ (<46.3% predicted) and PVR >16 Wood units had a particularly dismal prognosis, while patients with peak V’O_2_ ≥ 46.3% predicted and a PVR < 11.6 Wood units had >90% 5-year survival. Echocardiographic assessment of RV function in combination with CPET may provide incremental prognostic utility. Badagliacca and colleagues found that RV fractional area change on echocardiogram, in conjunction with the O_2_ pulse from CPET, which reflect RV function and stroke volume, were independent predictors of outcome in patients with idiopathic PAH (Badagliacca et al., 2016). Patients with RV fractional area change >26.5% and an O_2_ pulse >8.0 mL.beat^–1^ had excellent long-term survival, while PAH patients with RV fractional area change <36.5% and an O_2_ pulse <8.0 mL.beat^–1^ had significantly worse survival.

Others have also demonstrated that while V’_E_/V’CO_2_ slope as well as V’_E_/V’CO_2_ peak were associated with survival, once multivariate regression was performed, only ΔO_2_ pulse added prognostic value (Groepenhoff et al., 2008). Hemodynamic variables such as PVR and those that reflect right ventricular function (cardiac output, stroke volume, right atrial pressure) are also important predictors of prognosis in PAH (Saggar and Sitbon, 2012; Weatherald et al., 2018a,b; Benza et al., 2019). Wensel et al. evaluated the prognostic value of combining CPET-derived variables with haemodynamic data from RHC (Wensel et al., 2013) they assessed several CPET variables, including V’_E_/V’CO_2_, and found that only peak VO_2_, PVR, and HR change during exercise were independently associated with survival. Similarly, another study by Badagliacca et al., found that the only useful CPET parameter independently associated with future clinical worsening was peak VO_2_, with V’_E_/V’CO_2_ not adding additional prognostic information (Badagliacca et al., 2019).

## Conclusion

Cardiopulmonary exercise testing (CPET) is of great interest and utility for clinicians dealing with Pulmonary Hypertension (PH) in several ways such as: helping orienting diagnosis, evaluating exercise intolerance and its underpinning mechanisms, accurately assessing exertional dyspnea and unmasking its underlying often non straightforward mechanisms, generating prognostic indicators. Pathophysiologic anomalies in PH can range from reduced cardiac output and aerobic capacity, to inefficient ventilation, dyspnea, dynamic hyperinflation and locomotor muscle dysfunction. CPET can magnify the PH-related pathophysiologic anomalies and has a major role in the management of PH patients.

## Author Contributions

PL and JW equally contributed to the writing and revision of the manuscript. Both authors contributed to the article and approved the submitted version.

## Conflict of Interest

The authors declare that the research was conducted in the absence of any commercial or financial relationships that could be construed as a potential conflict of interest.
